# Circular polarization in a non-magnetic resonant tunneling device

**DOI:** 10.1186/1556-276X-6-101

**Published:** 2011-01-25

**Authors:** Lara F dos Santos, Yara Galvão Gobato, Márcio D Teodoro, Victor Lopez-Richard, Gilmar E Marques, Maria JSP Brasil, Milan Orlita, Jan Kunc, Duncan K Maude, Mohamed Henini, Robert J Airey

**Affiliations:** 1 Physics Department, Federal University of São Carlos, São Carlos, Brazil; 2 Physics Institute, UNICAMP, Campinas, Brazil; 3 Grenoble High Magnet Field Laboratory, Grenoble, France; 4 School of Physics and Astronomy, Nottingham Nanotechnology and Nanoscience Centre, University of Nottingham, Nottingham, NG7 2RD, UK; 5 EPSRC National Centre for III-V Technologies, The University of Sheffield, Sheffield, UK; 6 Institute of Physics, Charles University, Ke Karlovu 5, 121 16 Praha 2, Czech Republic

## Abstract

We have investigated the polarization-resolved photoluminescence (PL) in an asymmetric *n*-type GaAs/AlAs/GaAlAs resonant tunneling diode under magnetic field parallel to the tunnel current. The quantum well (QW) PL presents strong circular polarization (values up to -70% at 19 T). The optical emission from GaAs contact layers shows evidence of highly spin-polarized two-dimensional electron and hole gases which affects the spin polarization of carriers in the QW. However, the circular polarization degree in the QW also depends on various other parameters, including the *g*-factors of the different layers, the density of carriers along the structure, and the Zeeman and Rashba effects.

## Introduction

The understanding of the physics governing the dynamics of spin-polarized carriers in semiconductor structures is a fundamental issue for the development of new spintronic devices. In the past years, several systems have been proposed for spin-based devices, including magnetic metal/semiconductor junctions, all metallic devices, and all semiconductor systems [1-10]. However, the change of the polarization requires the use of an applied external magnetic field to change the contact magnetization. For some device applications, it would be interesting to have devices where the spin character of the injected or detected electrons could be voltage selected. One possible approach to achieve this goal is based on resonant tunneling diodes (RTDs) because the spin character of the carriers in the structure could be voltage controlled [11-15].

In this work, we have investigated the polarization-resolved photoluminescence (PL) from different regions in a non-magnetic asymmetric *n*-type RTD with a GaAs quantum well (QW) and AlAs and AlGaAs barriers. This asymmetry was used to increase the accumulation of charge of carriers in the QW. Under applied bias, electrons tunnel through the double-barrier structure creating a two-dimensional electron gas (2DEG) in the QW and at the accumulation layers next to the barriers which densities and *g*-factors are bias voltage dependent. The spin-dependent tunneling of carriers was studied by analyzing the current-voltage characteristics (*I*(*V*)) and the right (σ^+^) and left (σ^-^) circular polarized PL from the contact layers and from the QW under magnetic fields up to 19 T. High magnetic fields were used in order to increase the spin-related effects in our non-magnetic RTD. The main goal of the present work is to investigate the fundamental physics of spin-related effects in our structures, but this is an essential step for analyzing the feasibility of using RTD structures for spintronic devices in the future.

We have observed small oscillations on the QW circular polarization degree as a function of the applied voltage with values up to -70% at 19 T. We have also observed optical emission from spin-polarized 2DEG and two-dimensional hole gas (2DHG) in the GaAs contact layers next to emitter and collector barriers. Under applied bias voltage, polarized carriers from contact layer tunnel through the double-barrier region and contribute to the spin polarization of carriers in the QW. The circular polarization of the QW emission seems to depend on various other points, including the *g*-factors of the different layers, the spin-polarization of injected carriers from the contact region, the density of carriers along the structure, and the Rashba and Zeeman effects.

Our device was grown by molecular beam epitaxy on a *n*^+ ^(001) GaAs substrate. The double-barrier region consists of 2 μm *n*-GaAs (1 × 10^18 ^cm^-3^), 0.1 μm *n*-GaAs (1 × 10^17 ^cm^-3^), 51 Å undoped GaAs spacer, 40 Å AlAs barrier, 50.9 Å GaAs QW, 42 Å Al_0.4_Ga_0.6_As barrier, 51 Å GaAs spacer, 0.1 μm *n*-GaAs (1 × 10^17 ^cm^-3^), and 0.51 μm *n*-GaAs (1 × 10^18 ^cm^-3^). Circular mesas of 400 μm diameter were processed with annular AuGe contacts which allow optical measurements. Micro-PL measurements were performed at 4 K under magnetic fields up 19 T parallel to the tunnel current. The measurements were performed by using optical fibers and a Si CCD system coupled with a Jobin-Yvon spectrometer. A linearly polarized beam from an Ar^+ ^laser (all lines) was used for optical excitation. Therefore, photogenerated carriers in the structure do not present any preferential spin polarization. The right (σ^+^) and left (σ^-^) circularly polarized emissions were selected with appropriate optics and by reversing the current in the electromagnet.

**Figure 1:**
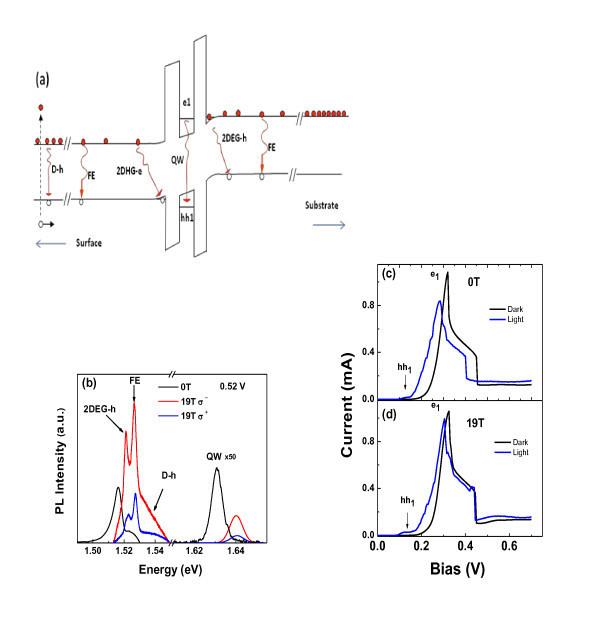
**Schematic band diagram of our device under forward bias, light excitation, and magnetic field parallel to the tunnel current (a)**. Typical PL emission from contact layers and QW region under 0.52 V and 19 T **(b)**. Current voltage characteristics curves for 0 and 19 T **(c,d)**.

Figure 1a shows a schematic band diagram of our device under forward bias voltage and light excitation. Under applied bias, a pseudo-triangular QW is created next to the emitter barrier. Electrons which occupy the quasi-bound states in the triangular QW form a 2DEG. Resonant tunneling can occur between 2DEG states in this triangular well and resonant states in the double-barrier structure (labeled e1). Photogenerated holes can also occupy the quasi-bound states in the triangular QW next to the top contact (collector barrier) and form a 2DHG. Therefore, resonant tunneling can also occur between 2DHG states and hole resonant states (hh1, lh1, and etc.) in the QW.

Under applied bias, photo-created holes can tunnel (resonantly or non-resonantly) and recombine with tunneling electrons into the QW and contact layers. The PL intensity from the QW is, in first approximation, proportional to the product of hole and electron densities. Therefore, it is very sensitive to the variation of charge density in the QW which can be voltage controlled in resonant tunneling devices. As a consequence, the PL intensity is, in general, correlated to the *I*(*V*) characteristic curves. In our experimental conditions, the optical emission from the QW was not detected under zero bias voltage, which indicates that the optical generation of carriers inside the QW is negligible.

Figure 1b shows typical polarization-resolved PL spectra for our device at 0.52 V. In general, the GaAs contact emission includes several bands: the free-exciton (FE) transition from the undoped space-layer, the recombination between photogenerated holes and donor related electrons from the *n*-doped GaAs layers (D-H), and the indirect recombination between free holes (electrons) and confined electrons (holes) localized at the 2DEG (or 2DHG) formed at the accumulation layer next to the barriers (2DEG-h or 2DHG-e emissions). In particular, only the 2DEG-h space-indirect emission was recently observed for *p*-*i*-*n *RTDs [13]. However, its voltage dependence and its contribution to the spin polarization of carriers in the QW were not investigated. In order to have more information about the contribution of both spin-polarized 2DEG and 2DHG to circular polarization degree of the QW emission, we performed a detailed measurement of the PL emissions as a function of the applied bias in our resonant tunneling structure.

Figure 1c shows the current voltage characteristics curve (*I*(*V*)) under dark and under light excitation at zero magnetic field. We have observed one electron resonant peak at *V*_1 _= 0.32 V which was associated with the resonant tunneling through the first confined electron state **e1** in the QW. Under light excitation, this resonant peak (**e1**) shifts to lower voltages. This shift is an evidence that the hole charge density has increased in the double-barrier region. Actually, an increase of the hole density in the QW reduces the total charge accumulated in the QW and shifts the electron resonant peak to lower voltages. The hole density at the accumulation layer also increases when we increase the applied voltage which results in the formation of a 2DHG next to the collector barrier (at the surface side, as illustrated in Figure 1a). As mentioned earlier, the energy position of confined levels in the 2DHG and QW can be voltage controlled. The hole resonant tunneling condition can be obtained by the alignment between the confined levels at the accumulation layer (2DHG) and QW. This effect is evidenced by the observation of an additional structure at 0.125 V in the *I*(*V*) curve under light excitation which was associated with the first heavy hole resonance (hh1) (Figure 1c). This additional structure hh1 is better defined under magnetic field (Figure 1d). In addition, we have observed that photocurrent under low voltages is markedly larger than the electron current in the dark, which indicates that the holes actually become the effective majority carrier under this voltage condition.

**Figure 2:**
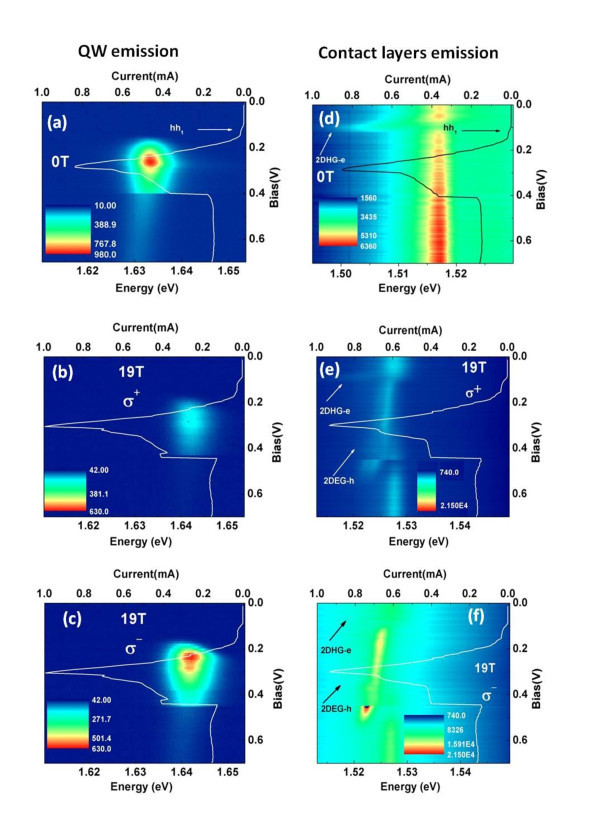
**Voltage dependence of PL for QW **(a)** and contact layers **(d)** under zero magnetic field and polarization resolved PL for QW **(b,c)** and contact layers **(e,f)** under 19 T**.

Figure 2a presents the voltage dependence of the QW PL at 0 T. The PL intensity increases in the electron resonance region (**e1**) and decreases after resonant tunneling condition. Therefore, the QW PL intensity presents a good correlation with the electron resonance which is due to the important increase of electron carrier density in the QW under resonant condition. As mentioned earlier, the PL intensity is proportional to the product of the hole and electron densities and, therefore, it is very sensitive to the variation of charge density in the QW (due to accumulated holes or electrons) which results in a modulation of the PL intensity near the resonant voltages. Figure 2b,c presents the voltage dependence of the QW PL intensity under 19 T for both σ^+ ^and σ^- ^polarizations. Under magnetic field, confined levels in the QW and contact layers split into spin-up and spin-down Zeeman states and the optical recombination can occur with well-defined selection rules giving information about the spin polarization of carriers in the structure. We have also observed a good correlation between the PL intensity and *I*(*V*) characteristic curve for both polarization σ^+ ^and σ^-^. We have also observed that the QW emission is highly σ^- ^polarized. The voltage dependence of the polarized degree of this emission will be discussed later in this manuscript.

Figure 2d-f presents the voltage dependence of the PL from the GaAs contact layers. As discussed earlier, this emission includes several bands: the FE transition, the recombination between photogenerated holes and donor-related electrons (broad band) and the voltage-dependent peaks which were associated with the indirect recombination between free electrons (holes) and the 2DHG (2DEG) at the accumulation layers (Figure 1a). The 2DHG-e emission is observed for low bias voltage, before the onset of hole resonant tunneling condition while the peak attributed to the 2DEG-h recombination is only observed under applied magnetic field, for bias voltages in the range of 0.1 <*V *< 0.5 V (Figure 2e,f).

We observed that an increase the applied bias results in a decrease of 2DHG-e emission intensity and in an increase of FE emission intensity. When we increase the applied voltage, photogenerated holes are swept away from the 2DHG by the increased electric field, and excitons are predominantly formed in the GaAs layer reducing the intensity of this 2DHG-e indirect transition. At hole resonant tunneling condition, holes tunnel through the QW which results in a reduction of hole density accumulated in the 2DHG which can also reduce the PL intensity of 2DHG-e emission. An abrupt transfer of FE emission to 2D electron - 3D hole emission was previously observed with increasing magnetic field (perpendicular to the 2DEG plane) for integer and fractional filling factors (ν < 2) on high quality modulation doped GaAs/AlGaAs heterojunction (HJ) [16-19]. Actually, it was observed that for filling factors ν < 2 the FE PL intensity decreases and a new lower energy PL line abruptly appears and gain intensity at expense of the exciton PL. This abrupt transfer was explained by a phenomenological dynamical model which considers an exciton dissociation near the magnetized 2DEG. In this model, the dissociation rate depends on exciton dynamics in two well potentials that is formed by FE near the HJ interface and 3D hole interacting with the 2DEG [18]. Our system is, however, more complex than a simple HJ structure. A variation of the applied voltage in the RTD results not only in strong variations of the carrier densities at the accumulation layers, and therefore variation of the filling factor of the 2D gases in the structure, but it also directly alters the electric field along the structure, and consequently, the potential profile *V*(*z*) at the accumulation layers. A complete analysis of the results requires detailed calculations, but we point out some general points which are consistent with our interpretation. The 2DHG-e peak is only observed before the onset of hole-tunneling, as for larger voltages the relatively small reservoir of photo-created holes (2DHG) accumulated at the top barrier interface must be mainly depleted. The 2DEG-h transition is observed at the electron resonant tunneling condition. Its intensity (Figure 2f) initially increases with bias voltage, which is consistent with increasing densities of tunneling holes and electrons accumulated at the 2DEG, but it shows some reduction at the **e1** resonance peak, as at this condition the density of the 2DEG should be somehow reduced. At about 0.45 V, when the *I*(*V*) characteristic curve shows an abrupt current reduction, the 2DEG-h shows an abrupt increase of intensity which is consistent with a sudden increase of electron density accumulated at the 2DEG. For large voltages (>0.5 V), the 2DEG-h emission tends to vanish, which may be associated with a reduced efficiency on the localization of holes around the 2DEG due to the significantly large electric field or to reaching a critical density of electrons at the 2DEG. As mentioned earlier, on the previous works on GaAs/GaAlAs HJs [16-19], the h-2DEG was only observed for magnetic fields larger than a critical value that corresponded to the filling factor ν = 2, which was pointed out as the limit case at which holes are still localized near the 2DEG. In our measurements, we maintained the magnetic field constant at 19 T. However, with increasing bias voltages, the density of electrons accumulated at the 2DEG should increase, and therefore, its filling factor should also increase. Therefore, it is possible that the condition ν ≥ 2 is attained for an applied bias voltage of about 0.5 V, resulting in the fading out of the 2DEG-h transition.

The observed 2DEG-h emission presents a high circular polarization degree with abrupt energy discontinuities after the electron resonance. This effect can be explained considering the increase of electron density in the accumulation-layer after the electron resonance and by changes in the overlap between the 2D electron and 3D hole wave functions induced by magnetic field which affects the 2DEG-h radiative recombination lifetimes of photo-excited holes [18,19].

**Figure 3:**
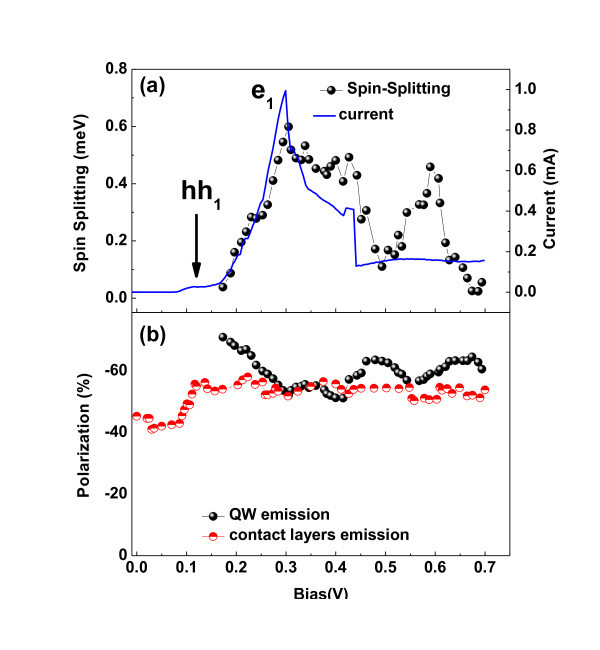
Voltage dependence of spin-splitting from QW emission **(a)** and circular polarization degree of contact layers and QW at 19 T **(b)**.

Figure 3 shows the voltage dependence of the excitonic spin splitting and circular polarization degree from the QW PL under 19 T. The circular polarization degree was calculated from the following relation: (*I*_σ+ _- *I*_σ-_)/(*I*_σ+ _+ *I*_σ-_); where *I*_σ+ _(*I*_σ-_) are the integrated intensity of the right (left) circular polarization. We have observed small oscillations on the voltage dependence of the polarization degree. The QW spin-splitting presents a small variation with applied bias probably due to the Rashba and Zeeman splitting tuning of hole levels by the effective electric field [12]. It was shown earlier that the electronic structure of RTDs are affected by the variation of the effective field in the double-barrier region and by modulation of Rashba SO and screening effects induced by hole charge buildup in the QW which results in a voltage modulation of spin-splitting [12]. However, we have observed that the circular polarization of the QW emission does not follow the measured spin-splitting energy of this emission. Therefore, it cannot be attributed to a simple thermal occupation effect of the QW excitonic states, which have a rather small effective *g*-factors. On the other hand, we observe that when we have a maximum in the excitonic spin-splitting we observed a minimum in the polarization degree. It seems that the excitonic spin splitting tends to change the sign of polarization degree of carriers in the QW. This effect could be explained if the *g*-factors of electrons and holes present opposite signs [20]. Under electron resonant condition the sign of polarization degree tends to be defined by the sign of *g*-factor of minority carriers (holes). In addition, we observe that, under higher voltages, the QW and contact layer emissions present similar values of polarization degree which indicates that carriers tunnel to the QW with a polarization degree previously defined in the contact layers. However, the quantitative voltage dependence on the QW polarization degree seems to be rather complex and probably involves other effects such as the alignment of the spin-split QW levels at the resonant condition, the spin polarization of electrons and holes in contact layers prior to their tunneling into the QW, assuming that they maintain their spin polarization during the tunneling process.

In conclusion, we have observed small oscillations on the polarization degree from the QW as a function of the voltage. We have evidence of highly polarized 2DEG and 2DHG in the RTD which can contribute to the polarization degree of carriers in the QW. The voltage dependence of the 2DEG-h emission under magnetic field presents some anomalies which can be explained by the voltage dependence of tunneling dynamics of carriers in the structure. Our results imply that the double-barrier structure creates a polarized two-dimensional gas with a strongly enhanced *g*-factor, which can act as a spin-polarized source of injected carriers in the structure. However, the circular polarization of carriers in the double-barrier region seems also to depend on various other points, including the *g*-factors of the different layers, the spin-polarization of carriers in the contact region, the density of carriers along the structure, and the Rashba and Zeeman effects in the valence band.

## Abbreviations

PL: photoluminescence; QW: quantum well; RTDs: resonant tunneling diodes; 2DEG: two-dimensional electron gas; 2DHG: two-dimensional hole gas.

## Competing Interests

The authors declare that they have no competing interests.

## Authors' contributions

LFS prepared figures and participated in the analyses of the data. YGG conceived of the study, carried out the PL and transport measurements, analyzed the data and wrote the paper. MDT prepared figures. VLR, GEM and MJSPB participated in the draft of the manuscript. MO and JK participate in the photoluminescence alignment and measurements. DKM is responsable for the transport setup. MH grown the RTD sample and RJA processed our RTD.
